# Public Awareness of Heart Failure Among the Population of Makkah City, Saudi Arabia: A Cross-Sectional Study

**DOI:** 10.7759/cureus.50504

**Published:** 2023-12-14

**Authors:** Faisal M Alzubaidi, Nasser Al Shanbari, Fawaz S Baalaraj, Muhanna M Almatrafi, Muath Alzahrani, Sultan A Alneefia, Mokhtar Shatla

**Affiliations:** 1 Department of Medicine and Surgery, College of Medicine, Umm Al-Qura University, Makkah, SAU; 2 Department of Community Medicine, College of Medicine, Umm Al-Qura University, Makkah, SAU

**Keywords:** awareness, public health, public knowledge, medicine, heart failure, heart disease, cardiology

## Abstract

Background

Heart failure (HF), a major public health problem worldwide, is a complex clinical syndrome caused by structural or functional heart disorders occurring when the heart cannot supply sufficient blood to the body. The most common cause of HF is impairment of the left ventricle. Public awareness of HF is critical for controlling the disease burden, recognizing disease severity, and determining its prognosis. Therefore, this study assesses the awareness and perception of HF among the population in Makkah City, Saudi Arabia.

Methods

A cross-sectional study was conducted among 1,053 participants over 18 years of age who lived in Makkah City between October 2022 and February 2023. Participants were randomly selected and recruited using a validated online questionnaire.

Results

Of the participants, 62.4% had heard of HF, and the majority (59.5%) correctly identified it. Regarding the etiology of the disease, about 50.6% indicated that it was related to the aging process, and 82.9% indicated it was related to high blood pressure. Only 24.1% of participants correctly recognized HF symptoms; most defined the symptoms as general heart disease. Moreover, 51.6% of participants agreed that the current HF medications can reduce deaths from HF and improve quality of life. However, most respondents disagreed that HF drugs can prevent the onset of HF.

Conclusion

The findings emphasize the need for more awareness programs to raise the public awareness about HF and effectively guide the population to more reliable sources that provide evidence-based information about the disease.

## Introduction

Heart failure (HF) is a complex clinical syndrome caused by a structural or functional cardiac disorder that results when the heart cannot provide the body with sufficient blood supply [1-3]. The most common cause of HF is when the left ventricular myocardial function is reduced, followed by the dysfunction of the pericardium, myocardium, endocardium, heart valves, or great vessels [4,5]. Further, HF is becoming a significant public health concern affecting an estimated 14 million Europeans [1,6]. According to the American Heart Association, over five million Americans over 20 have HF, and the estimated prevalence of HF in Saudi Arabia is 455,222 cases, with an estimated incidence of 32,200 cases annually [7]. Moreover, HF primarily results from injuries to the myocardium caused by a variety of factors, including ischemic heart disease, hypertension, and diabetes. Less common factors include cardiomyopathies, valvular disease, myocarditis, infections, systemic toxins, and cardiotoxic drugs [3].

A recent study in Saudi hospitals revealed that 20% of acute coronary syndrome admissions had HF [7,8], which can be classified based on the deficit site (as predominantly left ventricular, right ventricular, or biventricular) or according to the onset (as acute or chronic) [4,9]. Moreover, HF can be clinically classified based on the heart function status into two types: HF with preserved ejection fraction (EF) in those with a normal EF of over 50% and HF with a reduced EF of less than 40% [4,9].

The signs and symptoms of HF occur with inadequate cardiac output and poor blood supply. Primarily, the patients present with shortness of breath, coughing, wheezing, fatigue, weakness, lethargy, lower limb edema, and ascites [3,4,10]. For the diagnosis, various parameters are used with a physical examination to determine the presence of clinical signs and symptoms. Moreover, blood tests are employed, including a complete blood count, urinalysis, complete metabolic profile, blood urea nitrogen, serum creatinine, glucose, fasting lipid profile, liver function test, and thyroid stimulating hormone. Additionally, HF-specific laboratory tests are used, including brain natriuretic peptide (BNP) and N-terminal proBNP (which is more sensitive and less specific than BNP). Other diagnostic modalities include chest X-rays, transthoracic echocardiography, computerized tomography scans, and magnetic resonance imaging [4,5].

Other studies have demonstrated that over 40% of patients with HF die within the first year of their first hospitalization, indicating a poor prognosis for HF [6,11]. In this regard, public awareness of HF is critical for controlling the disease burden, recognizing disease severity, and determining its prognosis. However, studies worldwide on this topic are limited and primarily from Europe because there is a lack of information on public awareness of HF [12]. This study aims to assess the awareness and perception of HF among the population in Makkah City, Saudi Arabia.

## Materials and methods

This cross-sectional study was conducted between October 2022 and February 2023. The study includes participants over 18 who live in Makkah. Participants who refused to participate in the study were excluded.

The sample size was calculated using Open Epi software v2.1 (www.OpenEpi.com) [13]. The minimum calculated sample size was 486, considering a confidence interval of 95% and a significance level of 5%. However, 1,053 participants were recruited in this study to increase the efficiency of generalizing the study findings.

The Umm Al-Qura ethical committee granted this survey ethical approval in November 2022, with approval number HPAO-02-K-012-2022-11-1275, under the principles of the Helsinki Declaration. Participant names, phone numbers, and identity card numbers were not included to maintain anonymity and confidentiality. Before the survey, each respondent provided informed consent online and was made aware that the survey was voluntary, confidential, and only for academic purposes.

The study tool, an online questionnaire translated independently by two bilingual translators, was adapted from a previously published study [12]. The Arabic version was established, and a backward translation was conducted to compare the English version with the original to assess the accuracy of the questionnaire. Finally, a pretest was conducted by distributing the survey to 10 participants and then collecting their data and feedback. The questionnaire is divided into three sections. The first section collects demographic information. The second section contains questions that measure the study population’s awareness of HF before defining it, and the third section contains questions after providing the definition.

Statistical analysis was performed using Statistical Product and Service Solutions (SPSS, version 26) (IBM SPSS Statistics for Windows, Armonk, NY). The numerical data (including age only) are presented as the mean plus or minus the standard deviation. In contrast, the categorical data (including the rest of the variables) are presented as frequencies and percentages. A chi-square test was employed to assess the association between the sociodemographic characteristics of the participants and their knowledge of HF. Age was categorized as ≤30 or >30, and a p-value <0.05 was considered statistically significant.

## Results

A total of 1,053 participants who met the inclusion criteria completed the study questionnaire. The mean age of the participants was 33.5 (13.2) years, ranging from 18 to 70 years old. Additionally, 558 (53%) of the study participants were male, and 495 (47.0%) were female. The most predominant nationality of the participants was Saudi (n = 1006; 95.5%; Table 1).

**Table 1:** Sociodemographic characteristics

Demographics		Mean	SD
Age		33.5	13.2
		N (Total=1053)	%
Gender	Male	558	53.0
	Female	495	47.0
Nationality	Saudi	1006	95.5
	Other	47	4.5

The first three questions evaluated the awareness of the symptoms of cardiovascular disease: Question (Q) 1 for angina/myocardial infarction, Q2 for stroke, and Q3 for HF. Less than half of the participants answered correctly regarding Q1 (44.0%), Q2 (32.5%), and Q3 (24.1%). Moreover, Q3 was correctly answered much less than the two other diseases. When respondents were asked if they ever heard of HF, 62.4% answered that they heard of it. When the participants were asked to choose the best representation of HF, 59.5% chose "the heart cannot pump enough blood around the body.” When asked about the severity of the following symptoms: “breathlessness, tiredness, or swollen ankles,” only 37.0% recognized it as a serious illness. When they were asked, “How soon will you go to the hospital if you feel breathlessness, tiredness, or swollen ankles,” most (69.2%) answered that they would visit the hospital in one to two days, followed by within a week (18.0%), one to three weeks (6.4%), and a month (2.7%), and some (3.7%) would skip visiting the hospital. About one-third of the participants’ families suffer from heart disease, 13.8% from angina or myocardial infarction, 13.5% from HF, 12.0% from arrhythmia, and 22.0% from valvular heart disease (Table 2).

**Table 2:** Heart failure questions and their answers before giving the definition of heart failure

Question	Answer	N	%
What disease do you think of if someone has the following symptoms? Symptoms: chest heaviness and tightness that occur during exertion and disappearing with rest	Angina or myocardial infarction	463	44.0
	Gastrointestinal disorder	49	4.7
	Other heart diseases	291	27.6
	Lung disorders	156	14.8
	I do not know	94	8.9
What disease do you think of if someone has the following symptoms? Symptoms: facial paralysis, double vision, and/or sudden unilateral weakness in the arm	Stroke	342	32.5
	Parkinson's, epilepsy, and other brain disease	211	20.0
	Other heart diseases	109	10.4
	Angina or myocardial infarction	220	20.9
	I do not know	171	16.2
What disease do you think of if someone has the following symptoms? Symptoms: breathlessness with low-level activity, tiredness, and swollen ankles	Heart failure	254	24.1
	Heart in general	384	36.5
	Angina or myocardial infarction	73	6.9
	Lung disorders	178	16.9
	I do not know	164	15.6
Have you ever heard of heart failure?	Yes	657	62.4
	No	396	37.6
What do you think is the best representation of heart failure?	Heart having a blood- and oxygen-deprived state due to a clot formed in the vessel	240	22.8
	Heart rhythm abnormality	61	5.8
	Weakness of the heart due to the natural course of aging	49	4.7
	The heart cannot pump enough blood around the body	627	59.5
	I do not know	76	7.2
What do you think the severity is if you have the following symptoms: breathlessness, tiredness, or swollen ankles?	Serious illness	390	37.0
	Slightly serious illness	493	46.8
	Minor illness	56	5.3
	I do not know	114	10.8
How soon will you go to the hospital if you feel breathlessness, tiredness, or swollen ankles?	1-2 days	729	69.2
	Within 1 week	190	18.0
	1-3 weeks	67	6.4
	1 month	28	2.7
	Never (I will not go to the hospital)	39	3.7
Is there anyone among you or your family members who are suffering from heart disease?	Yes	400	38.0
	No	653	62.0
What is his/her diagnosis?	Angina or myocardial infarction	55	13.8
	Heart failure	54	13.5
	Arrhythmia (abnormality of heart rhythm)	48	12.0
	Valvular heart disease	88	22.0
	I do not know	155	38.8

Regarding the etiology of HF, about half of the participants (50.6%) answered that HF is part of the normal aging process. Regarding the contributing factors for developing HF, most respondents selected hypertension (82.9%), while the rest chose lung disorder (17.1%). Regarding the perception of HF-related risk, when the participants were asked about the lifetime risk of developing HF, only 18.3% answered correctly: “20 in 100 people.” When the participants were asked which of the following diseases had the highest mortality after five years of diagnosis, 44.3% chose HF, 26.5% indicated myocardial infarction, 22.4% chose stroke, and 6.8% selected prostate or breast cancer. Regarding the risk of sudden cardiac death in HF patients, 69.4% of the participants answered that they were worried about sudden cardiac death, and 12.2% were not worried about it. When the participants were asked about the HF risk of mortality within the first year of discharge, only 10.3% correctly answered, “20 in 100 people might die.” When the participants were asked about the readmission rate within a year after discharge from HF, 15.4% correctly selected “20 in 100 people.” The participants were asked to choose which disease has the greatest influence on quality of life, and the majority (61.1%) indicated HF, 19.0% selected diabetes, 13.0% chose hypertension, and 6.9% indicated arthritis.

As for HF treatment, most participants (48.5%) indicated that they would prefer a treatment that could improve their quality of life, and 19.2% preferred a treatment that would allow them to live longer. Their perceptions about HF medication were also investigated in the survey. About 51.6% agreed that HF medication can reduce death from HF. Further, 53.6% agreed that the current HF medications could improve the quality of life in patients with HF, and 35.1% agreed that current HF medications could prevent the occurrence of HF. When the participants were asked if they thought that HF patients should live quietly and reduce all physical activity, most (66.8%) answered “yes.” The preferred sources of HF-related information among the respondents were the internet (48.5%) and followed by primary healthcare (25.7%) and secondary or tertiary care clinics (23.6%; Table 3).

**Table 3:** Heart failure questions and their answers after the definition of heart failure is given

Question	Answer	N	%
Do you agree that heart failure is a normal aging process?	Yes	533	50.6
	No	520	49.4
Which of the following conditions is a precipitating cause for developing heart failure?	Hypertension	873	82.9
	Lung disorder	180	17.1
Which of the following do you think is the correct lifetime risk of developing heart failure?	1 in 100 people	262	24.9
	5 in 100 people	368	34.9
	10 in 100 people	230	21.8
	20 in 100 people	193	18.3
Which of the following diseases is most likely to have the highest mortality within 5 years after diagnosis?	Stroke	236	22.4
	Prostate cancer or breast cancer	72	6.8
	Heart failure	466	44.3
	Myocardial infarction	279	26.5
Suppose one of your friends, colleagues, or neighbors suffers from heart failure. Are you worried that they might suddenly die?	Yes	731	69.4
	No	128	12.2
	I do not know	194	18.4
Acute heart failure is a sudden worsening of the signs and symptoms of heart failure. Which of the following is correct for the post-discharge one-year mortality from acute heart failure?	2 in 100 people might die	206	19.6
	5 in 100 people might die	246	23.4
	10 in 100 people might die	140	13.3
	20 in 100 people might die	108	10.3
	I do not know	353	33.5
What is the readmission rate within one year after discharge from heart failure?	2 in 100 people	148	14.1
	5 in 100 people	236	22.4
	10 in 100 people	177	16.8
	20 in 100 people	162	15.4
	I do not know	330	31.3
What disease do you think will have the greatest impact on the quality of life? Please select only one.	Diabetes	200	19.0
	Arthritis	73	6.9
	Heart failure	643	61.1
	Hypertension	137	13.0
If you were a heart failure patient, which of the following treatments would you prefer?	Treatment that could improve the quality of life	511	48.5
	Treatment that allows you to live longer	202	19.2
	I cannot decide	340	32.3
Do you agree that "current heart failure medications could reduce death from heart failure"?	Yes	543	51.6
	No	99	9.4
	I do not know	411	39.0
Do you agree that "current heart failure medications could improve the quality of life in patients with heart failure"?	Yes	564	53.6
	No	101	9.6
	I do not know	388	36.8
Do you agree that "current heart failure medications could prevent the occurrence of heart failure"?	Yes	370	35.1
	No	241	22.9
	I do not know	442	42.0
Suppose one of your friends, colleagues, or neighbors suffers from heart failure. Do you think that they should live quietly and reduce all physical activity?	Yes	703	66.8
	No	154	14.6
	I do not know	196	18.6
If you need information about heart failure, where would you visit?	Primary care clinic	271	25.7
	Secondary or tertiary care clinic	249	23.6
	Oriental medicine clinic	0	0
	Pharmacy	22	2.1
	Internet	511	48.5

Table 4 lists the association between the answers to Q3 (“What disease do you think of if someone has the following symptoms? Symptom: breathlessness with low-level activity, tiredness, and swollen ankles”) and the sociodemographic characteristics. Most participants younger than 30 years (32.0%) and over 30 years (41.1%) answered that these are related to the heart in general, with a P value <0.000. Additionally, male (36.7%) and female (36.2%) respondents answered “heart in general,” with a P value of 0.639. Further, Saudi (36.1%) and non-Saudi respondents (44.7%) answered that these symptoms were more related to the heart in general, with a P value of 0.133.

**Table 4:** Association between question 3 (What disease do you think of if someone has the following symptoms? Symptoms: breathlessness with low-level activity, tiredness, and swollen ankles) answers and the sociodemographic characteristics

Demographics		Heart failure	Heart in general	Angina or myocardial infarction	Lung disorders	I do not know	P value
Age	≤30	161(29.8)	173(32.0)	37(6.9)	92(17.0)	77(14.3)	<0.000
	>30	93(18.1)	211(41.1)	36(7.0)	86(16.8)	87(17.0)	
Gender	Male	139(24.9)	205(36.7)	39(7.0)	85(15.2)	90(16.1)	0.639
	Female	115(23.2)	179(36.2)	34(6.9)	93(18.8)	74(14.9)	
Nationality	Saudi	249(24.8)	363(36.1)	70(7.0)	166(16.5)	158(15.7)	0.133
	Other	5(10.6)	21(44.7)	3(6.4)	12(25.5)	6(12.8)	

## Discussion

Although HF has a poor prognosis and that more than 40% of patients die within the first year of their first hospitalization, the awareness of HF in Europe remains unsatisfactory. Studies regarding the awareness among the Middle Eastern population are lacking as well (6,11,12). Therefore, this study assesses the awareness of HF among the population of Makkah City, Saudi Arabia. Accordingly, the findings revealed an acceptable level of awareness, whereas most participants have heard about HF (62.4%), and the majority defined it correctly (59.5%). Nevertheless, a substantial number of the respondents defined HF incorrectly. However, these results are significantly better than those in a Korean study, where only 47% could define HF correctly [12]. These results are consistent with the findings of the Europe (SHAPE) survey [6].

Regarding the etiology of the disease, around 50.6% indicated that it is related to the aging process. This misperception aligns with the opinions of one-third of the participants in the Korean study [12]. This finding indicates the need for more awareness programs to prevent the late diagnosis of the disease and the consequent poor outcomes. Moreover, 82.9% believed that HF is caused by hypertension.

According to primary care records in the UK, the five-year mortality of HF has been increasing to 48.5% [14]. Furthermore, US Medicare data report that mortality is 75% [15]. Despite that, the majority of the respondents did not recognize that HF has the highest mortality rate in contrast to MI, stroke, prostate cancer, and breast cancer. Only 18.3% recognized the correct lifetime risk of developing HF, which the Framingham Heart Study reported as 20%, regardless of sex [16].

When HF was identified with the symptoms of heart conditions in general, the Korean population exhibited better awareness, where 62% correctly recognized HF [12]. This finding indicates the need for more educational programs and campaigns to improve the awareness of the population regarding HF. Conversely, most participants stated that HF was a serious illness.

More than half of the participants agreed that the current HF medications could reduce death from HF and improve the quality of the patients’ lives. This state of awareness is beneficial for the acceptance and tolerance of medications and should be enhanced in the rest of the population. However, most respondents disagreed that HF medications could prevent the occurrence of HF.

Two-thirds of the Korean population preferred secondary and tertiary care clinics as sources of information about HF [12]. In contrast, the study participants preferred the internet as a source of information regarding the disease. This preference demonstrates the necessity of effectively guiding the population to more reliable sources that provide evidence-based information about the disease.

A few limitations exist in this study. A more randomized sampling technique should be used for more accurate results. The variations in the number of population categories affect the generalization accuracy of the study findings. Therefore, the variation should be minimized in further studies of the same design.

## Conclusions

The focus of this study was to assess the awareness and perception of HF among the population in Saudi Arabia. Most participants had heard of HF, and the majority correctly defined it. However, multiple flaws exist in their understanding of numerous aspects of the disease, requiring more educational programs and campaigns guided by specialized physicians to improve their knowledge comprehensively. The preferred source of HF information was the internet, highlighting the need to effectively guide the population to more reliable sources that provide factual and scientifically proven information about the disease.
